# Retraction Note: TMED3 promotes cell proliferation and motility in breast cancer and is negatively modulated by miR-188-3p

**DOI:** 10.1186/s12935-024-03310-8

**Published:** 2024-04-05

**Authors:** Jing Pei, Jing Zhang, Xiaowei Yang, Zhengsheng Wu, Chenyun Sun, Zhaorui Wang, Benzhong Wang

**Affiliations:** 1 Department of Breast Surgery, Department of General Surgery, The First Affiliated Hospital of Anhui Medical University, Number 218, Jixi Road, Hefei, 230022 Anhui People’s Republic of China; 2 Department of Breast Surgery, The Tumor Hospital of XuZhou, HuanCheng Road 131, Xuzhou, 221003 Jiangsu People’s Republic of China; 3 Department of Pathology, The First Affiliated Hospital of Anhui Medical University, Jixi Road 218, Hefei, 230022 Anhui People’s Republic of China

**Retraction Note to: Cancer Cell Int (2019) 19:75**

10.1186/s12935-019-0791-4

The Editors-in-Chief retracted this article because of concerns regarding a number of figures presented in this work. These concerns call into question the article’s overall scientific soundness. An investigation conducted after its publication discovered similarities between images in this work:

- The panel labelled sh-control in Fig. 3C overlaps with the panel labelled sh-control in Fig. 3D, after a 90º rotation.
- The Beta-actin band in Fig. 4G overlaps with a portion of the Beta-actin band in Fig. 2G, after a horizontal flip and a change in scale.

The Editors-in-Chief therefore no longer have confidence in the integrity of the research presented in this article. The authors have not replied to correspondence from the Publisher.

